# Toward Complementary Characterization of the Chemical Bond

**DOI:** 10.1021/acs.jpclett.2c02544

**Published:** 2022-10-27

**Authors:** Maciej Hendzel, Maciej Fidrysiak, Józef Spałek

**Affiliations:** Institute of Theoretical Physics, Jagiellonian University, ulica Łojasiewicza 11, PL-30-348 Kraków, Poland

## Abstract

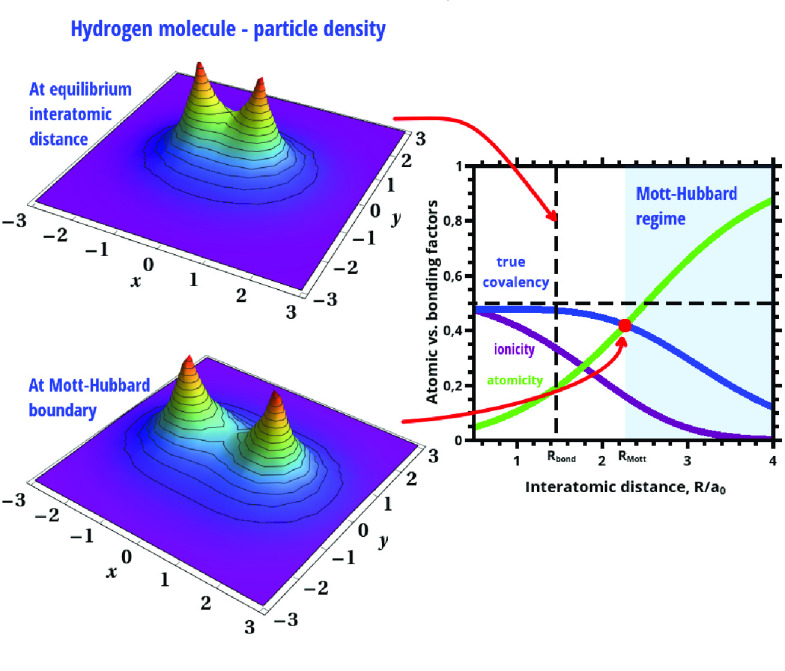

A precise discussion of a single bond requires consideration of two-particle wave function for the particles involved. Here we define and determine rigorously the intrinsic covalency and connected characteristics of the canonical example of the H_2_ molecule. This is achieved by starting from an analytic form for the two-particle wave function for electrons forming the bond, in which we single out the atomic contribution (*atomicity*) in an unequivocal manner. The presence of the *atomicity* and ionicity factors complements the existing attributes of the bond. In this way, a gradual evolution of the molecular state to its two-atom correspondent is traced systematically with increasing interatomic distance. In effect, a direct relation to the onset of incipient Mott-Hubbard atomicity (*Mottness*) to the intrinsic covalency and ionicity is established. This goal is achieved formally by combining the single-particle wave function readjustment in the entangled state with a simultaneous determination of two-particle states in the particle (second quantization) representation.

The concept of a chemical bond and its quantum properties is of fundamental importance to our understanding of both physical and chemical characteristics of molecules and solid-state compounds.^1−3^ Among the principal questions are those of the relative roles of covalency, ionicity, and atomicity, as they describe qualitative differences with the characteristics of parent atomic states composing the system. Historically, the chemical bond was described in a quantitative manner by Heitler and London^4^ (see also Condon^5^)—who introduced the two-particle wave function in the Hartree–Fock approximation that is composed of atomic wave functions of electrons centered on atoms. This inconsistency in an otherwise pioneering approach needed refinement to include a quantum-mechanical mixing of the atomic orbitals.^6−8^ In effect, the approach is a complete single-particle formulation of the intrinsic many-particle problem.

Here we propose a resolution of the question concerning the evolution of the molecular states into the corresponding atomic configuration within the many-particle picture. Such an approach leads to an unequivocal determination of *true covalency*, incorporating a novel concept of *atomicity*, in addition to the *ionicity*. In this way, we resolve the longstanding fundamental question of unphysical behavior of covalency with the increasing interatomic distance as well as observe a gradual evolution of the correlated molecular Hund–Mulliken (Hückel–Slater) orbitals into their Heitler–London atomic correspondents with the increasing interatomic distance, *R* → *∞*.

At the outset, we take a multiparticle view of the chemical bond and implement a special method EDABI (**E**xact **D**iagonalization **Ab I**nitio approach) devised in our group earlier^9−11^ and apply it here to a rigorous analysis in the simplest situation of the H_2_ molecule. The generic case of the H_2_ molecule involves a two-electron single bond, and thus the role of interelectronic correlations in combination with concomitant molecular single-particle wave-function readjustment in the resultant (correlated) state can be analyzed rigorously and in mathematically analytic terms.

Our formulation differs essentially from the discussion concerning, among others, the role of electron correlations in the chemical bonding,^12−19^ as neither the *atomicity* nor *intrinsic covalency* factors are singled out or even discussed explicitly. The introduction of *atomicity* is indispensable to obtain correct bonding characteristics behavior, as discussed in detail below. In this sense, we introduce a complementary description in the quantum-mechanical language, as it contains both “delocalized” (molecular) and “localized” (atomic) ingredients to complete the picture.

The structure of this Letter is as follows. After introducing an exact two-particle electron wave function, we redefine the covalency to extract from the standard definition the contribution of atomicity, by referring to the notions of Mott and Hubbard localization. This new formulation allows us to define and determine explicitly the true covalency, atomicity (also termed seniority^20^), and ionicity factors in the chemical bond and hence to understand those component factors of the chemical bond in a fully quantitative manner. The whole methodology is based on a combination of both the first- and second-quantization aspects of the relevant multielectron states, detailed in the Supporting Information.

By applying the procedure outlined in the Method and in Supporting Information we obtain a two-particle wave function in an explicit form
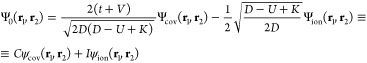
1where the covalent (Ψ_cov_) and ionic (Ψ_ion_) parts are, respectively
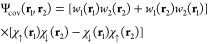
2
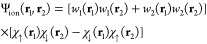
3where  are trial Hückel-Slater molecular orbitals and  are spin functions. In the above expression the microscopic parameters *U* and *K* are the magnitudes of intra-atomic and interatomic Coulomb repulsion, respectively, *t* and *V* are the magnitudes of hopping (Bloch integral) and so-called correlated hopping, respectively (for details see Methods), and
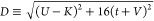
4

We note that the two-particle wave function has the Heitler–London form, except the coefficients contain **all** interparticle interaction terms, and the single-particle wave functions are the molecular Hückel-Slater orbitals with their adjusted size in the resultant correlated state. This two-particle wave function may be rewritten in terms of the original Slater orbitals as follows
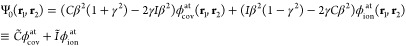
5where the coefficients β and γ are defined through the relation
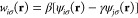
6where *i* and *j* label the atoms, and σ ≡ ±1 ≡ *↑*, *↓* is the electron spin quantum number. Note that ⟨*w*_*i*_(**r**)|*w*_*j*_(**r**)⟩ = δ_*ij*_, β, and γ are the mixing coefficients of the neighboring Slater orbitals (), (in which α^–1^ is the size of the orbital). The functions  and  have the same form as (2) and (3), respectively, except for the replacement *w*_*iσ*_(**r**) → ψ_*iσ*_(**r**).

We define the effective atomic contribution  at given interatomic distance *R* as
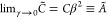
7that is, we regard it as the weight of the atomic part at the same *R*. Formally, the true covalency, ionicity, and atomicity are then defined for a given *R* as
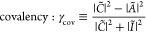
8
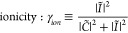
9
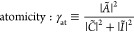
10

The original motivation for introducing the concept of atomicity is as follows: It may seem natural to define covalency as |*C*|^2^/(|*C*|^2^ + |*I*|^2^) and ionicity as |*I*|^2^/(|*C*|^2^ + |*I*|^2^). However, such a choice leads directly to unphysical features (see Supporting Information). Namely, |*C*|^2^/(|*C*|^2^ + |*I*|^2^) reaches its maximal value of unity in the limit of separate atoms (*R* → *∞*). This is also the limit when electrons are entirely localized on their parent atoms and become distinguishable in the quantum-mechanical sense. This is the limit that we regard as that atomicity equal to unity and vanishing true covalency. This type of argument is also the reason for subtracting the probability  from , not the corresponding wave–function amplitudes. The quantities (8)–(10) are proved next to be useful and of crucial importance.

To demonstrate the fundamental meaning of the introduced quantities we relate them to the criteria of Mott^21^ and Hubbard^22^ for onsets of localized (atomic) behavior. This is because the evolution of molecular H_2_ (electron-paired) state into individual separate singly occupied (atomic) states is regarded as equivalent to the Mott–Hubbard localization (for a recent related discussion in different context see, e.g., ref (23)). Namely, we define Mott and Hubbard onset criteria as
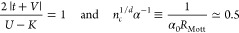
11respectively, where α_0_ is the readjusted inverse orbital size, here at *R* = *R*_Mott_. The first of them implies that, for *R* = *R*_Mott_, the kinetic (hopping) energy is equal to the correlation energy; that is, for *R* < *R*_Mott_ the ratio is greater than unity, whereas for *R* > *R*_Mott_ it is smaller than unity and reduces quite rapidly to zero with increasing *R* beyond *R*_Mott_. In other words, the kinetic energy dominates in the former case and enhances hopping electrons to resonate strongly between the sites, whereas the electrons become gradually frozen as *R* increases beyond *R*_Mott_. On the other hand, the Mott criterion expresses the onset of localization in terms of the renormalized single-particle wave function size at the localization threshold. Namely, the threshold is reached when the diameter of the orbital in the correlated state  is equal to the interorbital distance (*R* = *R*_Mott_). Semiclassically, it means that the collective character is established when the orbitals start overlapping.

To visualize this formal reasoning we have plotted in Figure 1 the left parts of both (11) as a function of *R* as well as have marked by red points their values at *R*_Mott_. The blue shaded area may be called *the Mottness regime*.^24^ The inset shows the corresponding dependence of the renormalized size of the Slater orbital in the correlated state, with the dotted vertical line marking the equilibrium bond length *R*_bond_ ≃ 1.43*a*_0_. Note that the orbital size for *R* > *R*_Mott_ approaches rapidly to the free-atom values *a*_0_.

**Figure 1:**
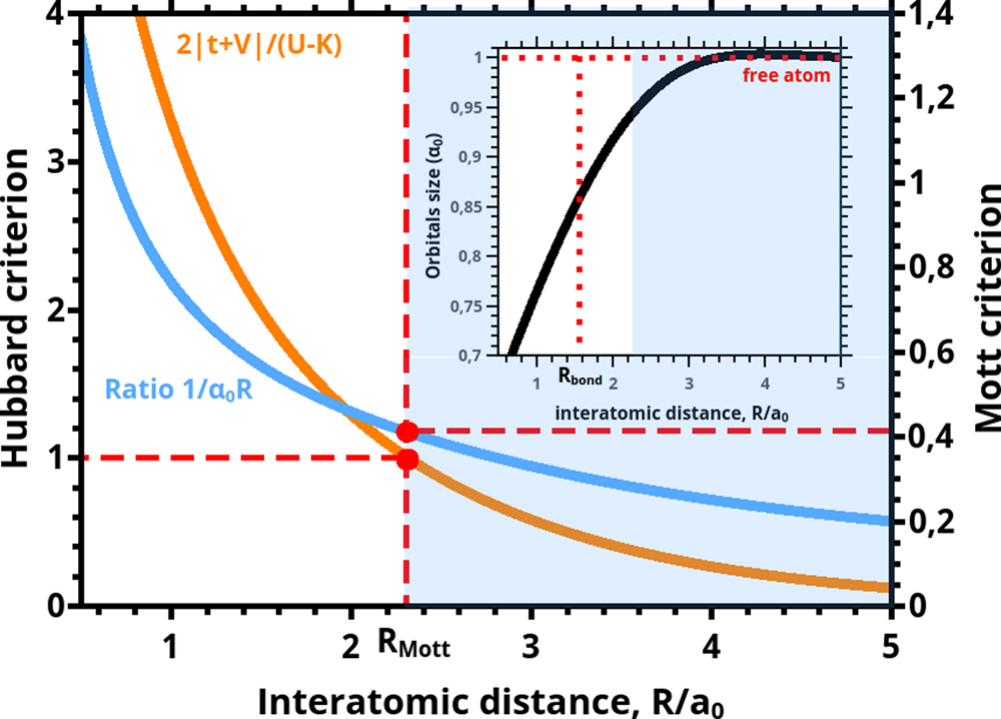
Hubbard (orange) and Mott (blue) characteristics of atomicity vs interatomic distance *R* (dashed horizontal lines). The dots mark the points corresponding to Hubbard and Mott criteria. The vertical dotted line marks the onset of *Mottness* at *R*_Mott_. (inset) *R* dependence of the orbital size of the renormalized atomic wave functions composing the molecular (Wannier) single-particle states. The dotted line marks the equilibrium distance *R*_bond_.

The explicit connection of the above onset to the true covalency (γ_cov_), ionicity (γ_ion_), and atomicity (γ_at_) is visualized in Figure 2, where the *R* dependence of those quantities is drawn. Remarkably, at the distance *R*_Mott_ the true covalency and atomicity acquire the same value, so the point *R*_Mott_ is a crossover point from true-covalency dominated to atomicity (*Mottness*) regime. Furthermore, γ_cov_ is predominant for *R* < *R*_Mott_, whereas γ_at_ is for *R* > *R*_Mott_, as it should. Additionally, the covalency and ionicity (atom double occupancy) coincide as *R* → 0, whereas then γ_at_ → 0. Both γ_cov_ and γ_ion_ disappear in the atomic limit (*R* ≫ *R*_Mott_), where γ_at_ → 1. The results presented in Figure 2 illustrate one the central findings of the present work. The principal characteristics are detailed further in Tables 1 and 2. In Table 1 we list the discussed factors of Mottness onset to show that they are mutually consistent. This agreement leads to the conclusion that the introduced entities (8)–(10) are not only relevant for the description of the Mott–Hubbard localization in condensed-matter (extended) systems but also appear as a crucial incipient feature in molecular systems. We stress, this was possible only by introducing a two-particle wave function as the proper characteristic of a single bond, which, after all, is composed of electron pairs.

**Figure 2:**
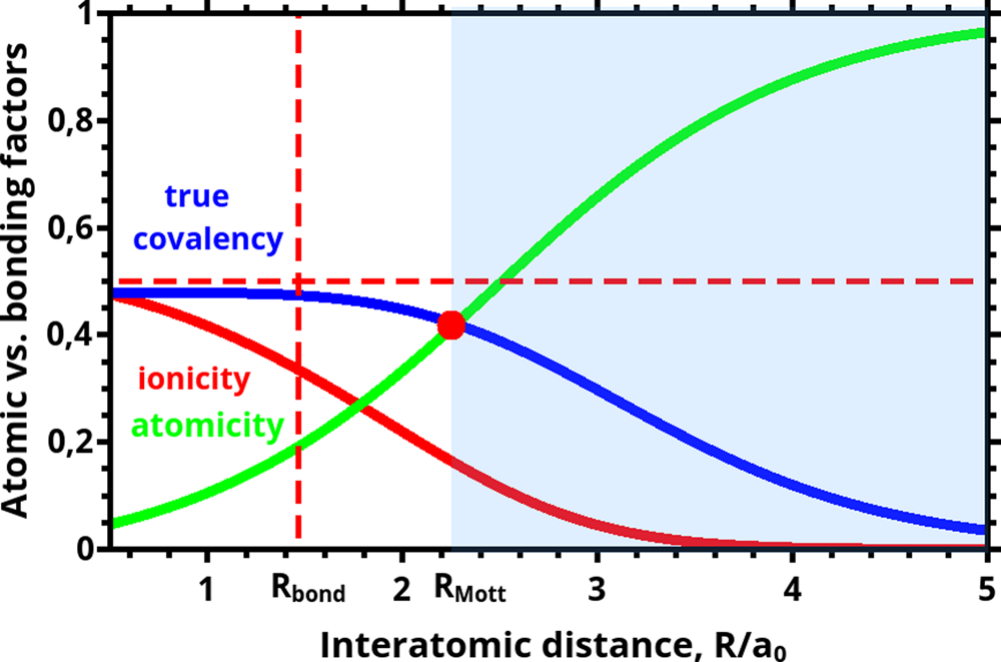
Intrinsic properties of the chemical bond: atomicity (green), true covalency (blue), and ionicity (red), all as a function of interatomic distance *R*. They represent the relative weights in the total two-particle wave function. In the *R* → 0 limit the atomicity practically disappears and is the only contribution in the separate-atom limit *R* → *∞*. The solid circle defines the onset of localization effects (*Mottness*) due to interelectronic correlations. If the atomicity is disregarded, the covalency exhibits a drastic nonphysical behavior with increasing *R* > *R*_bond_. The figure illustrates a systematic evolution of molecular states into separate atoms and, vice versa, the formation of molecular states out of separate atoms. The Slater states have a renormalized size α^–1^ ≤ α_B_.

**Table 1:** Equivalent Characteristics of the Atomicity Onset Threshold^a^

characteristic	value
Mott criterion	0.42
Hubbard criterion	1
covalent-atomic crossover	2.285*a*_0_
*R*_Mott_	2.279*a*_0_

a For details see the main text.

**Table 2:** Particles Density at the Midpoint, Inverse Orbital Size α_0_, and Mixing Coefficients β and γ, all Versus *R*^a^

*R* (*a*_0_)	*n*(0, 0)	α_0_	β	γ
1	0.334	1.307 51	1.1386	0.478 11
1.43	0.267	1.198 38	1.0854	0.388 77
2.3	0.142	1.054 28	1.0338	0.253 58
4	0.023	0.998 601	1.0012	0.048 640

a The particle density *n*(0, 0) illustrates the gradually vanishing electron density in the region between the atoms as *R* increases beyond *R*_Mott_.

So far, our discussion was based on wave-function mechanics. In the remaining part we reformulate the analysis directly in the second-quantization language, which will allow us to provide the physical interpretation of the bond in terms of particle densities. Namely, to amplify our multiparticle bond description we return to the particle language and display in Figure 3 several panels composed of electron density in the (*x*, *y*) plane, with the protons distant by *R*/*a*_0_ = 1, 1.43 (*R*_bond_), 2.3 (*R*_Mott_), and 4 (profiles a–d)), respectively. The density is defined as
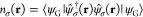
12where |ψ_G_⟩ is the lowest spin-singlet eigenstate and  is the field operator. One should note that this density when integrated and summed over spin directions (σ ± 1) is equal to the total number of particles (*N*_e_ = 2). Obviously, *n*_*↑*_(**r**) = *n*_*↓*_(**r**) ≡ *n*(**r**)/2, where *n*(**r**) is the total density. More importantly, this quantity provides the physical density, in contrast to the probability density |ψ_G_(**r**_1_, **r**_2_)|^2^. This distinctive feature of *n*(**r**) shows that the density diminishes in the region between the atoms to zero relatively fast with the increasing *R* above *R*_Mott_. To substantiate the last statement we have listed in Table 2 the density value *n*(0, 0) in the middle point between the proton positions. For the sake of comparison, we have also added there the inverse orbital size as well as the mixing coefficients in the wave function *w*_*i*_(**r**) = *w*(**r** – **R**_*i*_) to show that, indeed, both two- and single-particle characteristics merge into their atomic correspondents as *R* increases beyond *R*_Mott_. Note that, whereas γ expresses the decreasing Pauling covalency,^25^*n*(0, 0) describes the diminishing true covalency.

**Figure 3:**
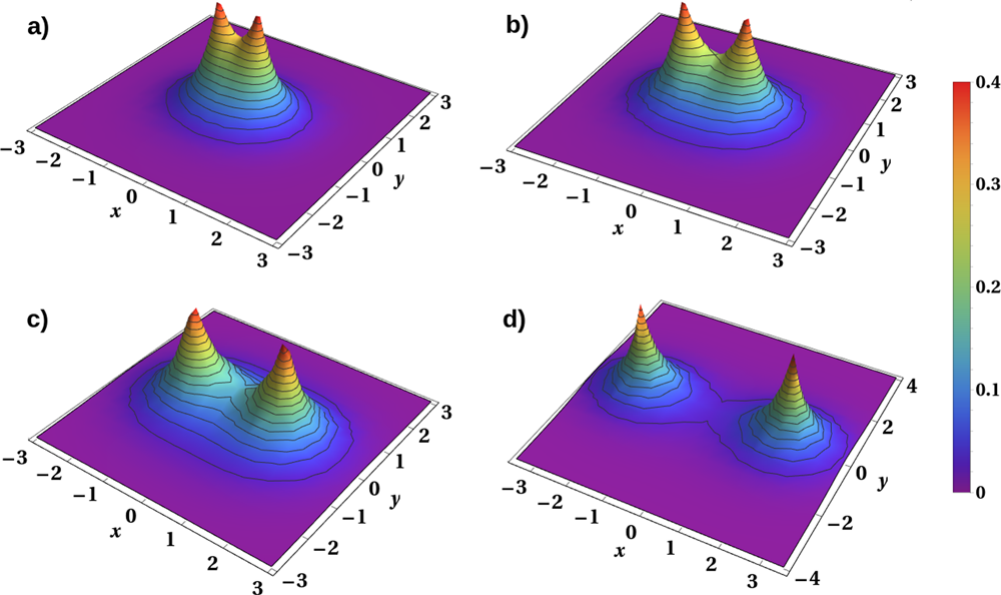
Electron density *n*_σ_(**r**) for different interatomic distances. (a) *R* = 1*a*_0_^–1^, (b) *R* = 1.43*a*_0_^–1^, (c) *R* = 2.3*a*_0_^–1^, (d) *R* = 4*a*_0_^–1^. The parts centered at nuclei are practically disjoint for *R* ≥ 5*a*_0_, illustrating the robustness of atomic behavior in that situation. This density contains also the double-occupancy (ionicity) contribution, which becomes rapidly negligible with increasing *R* beyond *R*_Mott_.

Analogously, we can define the concentration of the electrons in an ionic configuration as follows
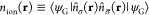
13with  and σ̅ ≡ −σ. In other words, *n*_ion_(**r**) expresses the density of local spin-singlet pairs (double-site occupancy). Profiles of *n*_ion_(**r**) are not presented explicitly, as they are contained in the equivalent form as the second part of the wave function (5).

Finally, along with the definitions (12) and (13) of local particle densities, we can define the nonlocal density of covalent electrons in the following manner.
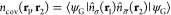
14This expression for a correlation function completes our description in both the first- and second-quantization schemes. The expressions (12)–(14) may be useful in the situations with more involved orbitals.

To recap, our analysis of a single chemical bond is based entirely on the multiparticle description, both in the first- and second-quantization schemes. Both of these descriptions are equivalent, but within the second part of them it is possible to relate it directly to the particle language. In general, the approach provides a precise revision of the bond factors as well as complements the current intense discussion^12−19^ of the correlation effects in the chemical bond description with the indispensable factor—the atomicity. Also, the method bridges the atomic and molecular aspects of the chemical bond in precise multiparticle categories. A similar analysis may be carried out for the evolution of covalent into ionic bonds^26^ in a more complex situation. Namely, by extending the single-particle basis in definition (16) of the field operators with, for example, 2p, etc. states, one can deal with more complex (e.g., C–C) bonds. In that situation, the subsequent analysis is still exact and feasible, but it is fully numerical in that case. Instead, one of our aims here was to exhibit the novel concepts/features of the problem in practically analytical terms. We should see a progress along these lines in the near future. In general, our description uses complementary pictures in the quantum-mechanical sense (molecular vs atomic).

*Method*. Our analysis starts from the full form of a Hamiltonian in the second quantization, with all interaction terms between electrons on the lowest orbitals, that is
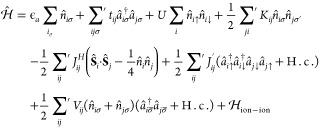
15where H.c. denotes the Hermitian conjugation,  are Fermionic annihilation (creation) operators for state *i* and spin σ, , and . The spin operators are defined as  with {σ_*i*_^*ab*^} representing Pauli matrices. The Hamiltonian contains the atomic and hopping parts (∝ϵ_*a*_ and *t*_*ij*_, respectively), the so-called Hubbard term ∝ *U*, representing the intra-atomic interaction between the particles on the same atomic site *i* with opposite spins, the direct intersite Coulomb interaction ∝ *K*_*ij*_, Heisenberg exchange ∝*J*_*ij*_^H^, and the two-particle and the correlated hopping and intersite Coulomb terms (∝*J*_*ij*_^′^ and *V*_*ij*_, respectively). The last term describes the ion–ion Coulomb interaction, which is adopted here in its classical form. The microscopic parameters (ϵ_*a*_, *t*_12_ ≡ *t*, *U*, *K*_12_ = *K*, *J*_12_^H^ = *J*_12_^′^, and *V*_12_ = *V*) are all calculated explicitly in the resultant correlated state by readjusting the single-particle wave function size contained in their expressions (for their analytic expressions see Supporting Information). The primed summations are taken for *i* ≠ *j*. The evolution of the new introduced quantities: atomicity, true covalency, and ionicity, is analyzed in detail as a function of interatomic distance.

The Hamiltonian 15 was determined by defining first the field operators  and , that is
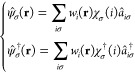
16where  are the annihilation (creation) operators of the single-particle states *w*_*i*_(**r**)χ_σ_(*i*) on atom *i* = 1, 2 with spin σ. Note that the single-particle basis is composed of the Hückel-Slater orbitals only; this restriction represents the only approximation here; the whole subsequent analysis and results are exact within these limitations. Those operators, in turn, lead to the expression (15) of Ĥ in a standard manner. To close the formal methodological part, the two-particle spin-singlet wave function is defined as^27^
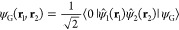
17where |0⟩ is the universal vacuum state for particles and |ψ_G_⟩ is the ground state, both in Fock space. This relation provides an equivalence of description both in terms of the two-particle wave function and the second-quantization language.

In Figure 4 we provide the flowchart of the numerical part of our analysis, which concerns mainly the determination of the orbital size α^–1^ in the interacting (correlated) state and, in effect, of the optimal ground-state energy and all other bonding-state characteristics. The numerical sampling of the variational minimization with respect to α (inverse readjusted atomic-orbital size) is carried out by taking the step Δα = 0.01. Further step diminishing does not influence significantly numerical values. The solution is exact with its method implemented in *Python* and *C++* languages, using additionally the *QMT* library.^28^

**Figure 4:**
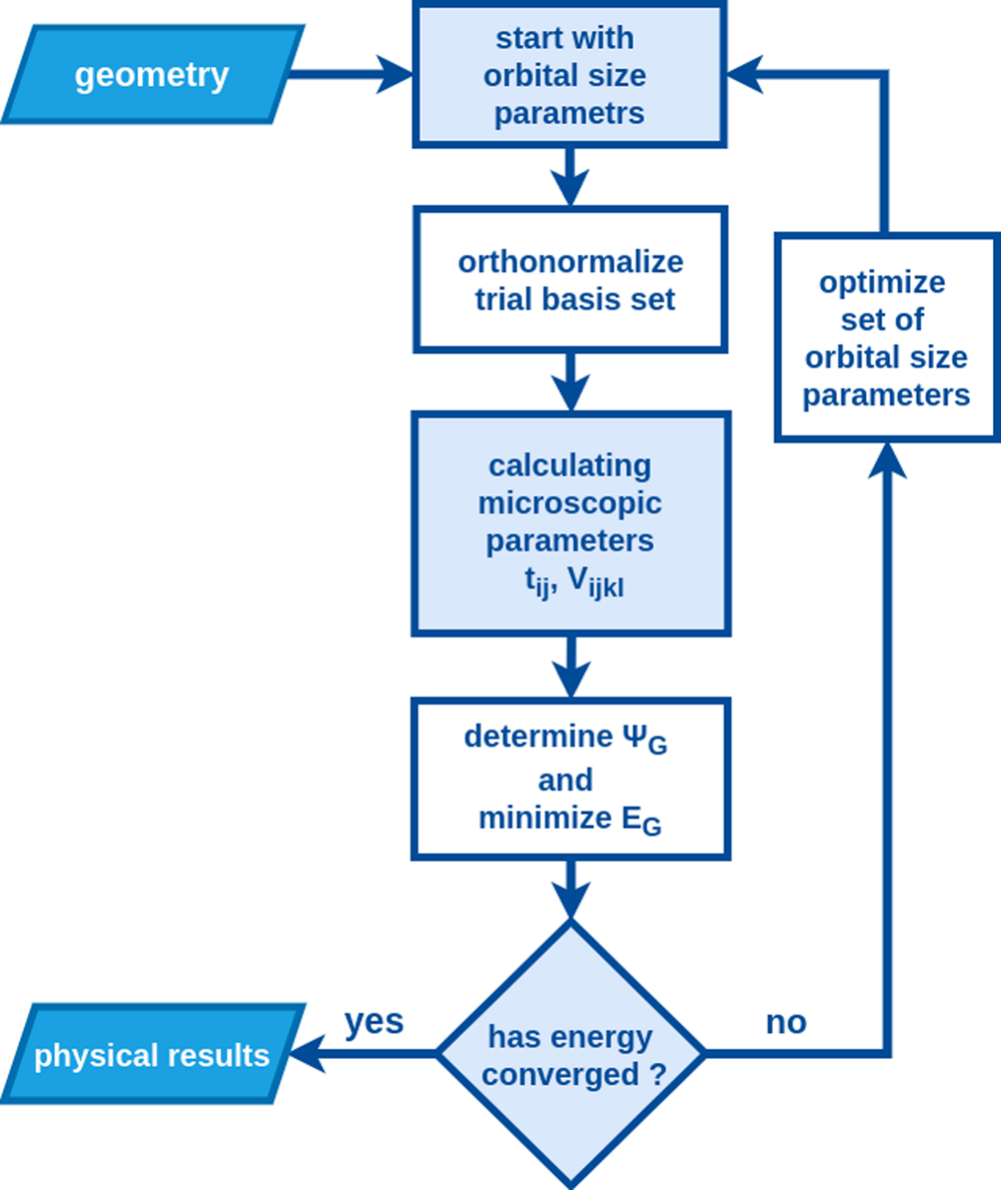
Flowchart of the EDABI method. The method is initialized by selection of a trial single-particle basis of wave functions (6) and subsequent diagonalization of the many-particle Hamiltonian (15). Optimization of the single-particle-state size leads to an explicit determination of the trial-wave function parameters, microscopic interaction and hopping parameters as well as ground-state energy, and explicit form of the many-particle wave function, all in the correlated interacting state for a given interatomic distance.

## Supporting Information Available

Additional methodological details, including step-by-step analytic calculations (PDF)
Transparent Peer Review report available (PDF)
