# Supplementary material for: Toward Complementary Characterization of the Chemical Bond
Source: J Phys Chem Lett. 2022 Oct 27;13(44):10261–6. doi: 10.1021/acs.jpclett.2c02544 (PMC9661532; doi:10.1021/acs.jpclett.2c02544)
Supplement: Supplementary file 1 — jz2c02544_si_002.pdf [file jz2c02544_si_002.pdf]

Supporting Information: Towards Complementary Characterization of the Chemical Bond

Maciej Hendzel, Maciej Fidrysiak, and Józef Spałek*

Institute of Theoretical Physics, Jagiellonian University, ulica Łojasiewicza 11, PL-30-348 Kraków, Poland

E-mail: jozef.spalek@uj.edu.pl

The formal solution of Hamiltonian (cf. Eq. (15) in main text) is carried out explicitly by selecting a trial basis in the Fock space for this two orbital systems. In the case with $N = 4$ spinorbitals and $N_e = 2$ electrons we have $\binom{N}{N_e} = \binom{4}{2} = 6$ states in the occupation number representation. They are

$$\begin{cases} |1\rangle = \hat{a}^\dagger_{1\uparrow}\hat{a}^\dagger_{2\uparrow}|0\rangle\,, \\ |2\rangle = \hat{a}^\dagger_{1\downarrow}\hat{a}^\dagger_{2\downarrow}|0\rangle\,, \\ |3\rangle = \frac{1}{\sqrt{2}}(\hat{a}^\dagger_{1\uparrow}\hat{a}^\dagger_{2\downarrow} + \hat{a}^\dagger_{1\downarrow}\hat{a}^\dagger_{2\uparrow})|0\rangle\,, \\ |4\rangle = \frac{1}{\sqrt{2}}(\hat{a}^\dagger_{1\uparrow}\hat{a}^\dagger_{2\downarrow} - \hat{a}^\dagger_{1\downarrow}\hat{a}^\dagger_{2\uparrow})|0\rangle\,, \\ |5\rangle = \frac{1}{\sqrt{2}}(\hat{a}^\dagger_{1\uparrow}\hat{a}^\dagger_{1\downarrow} + \hat{a}^\dagger_{2\downarrow}\hat{a}^\dagger_{2\uparrow})|0\rangle\,, \\ |6\rangle = \frac{1}{\sqrt{2}}(\hat{a}^\dagger_{1\uparrow}\hat{a}^\dagger_{1\downarrow} - \hat{a}^\dagger_{2\downarrow}\hat{a}^\dagger_{2\uparrow})|0\rangle\,. \end{cases} \tag{S1}$$

The first three are the spin–triplet states, whereas the next three are the spin–singlets and the mixture of those will result in the ground state. By calculating the Hamiltonian matrix

$\langle i| \hat{\mathcal{H}} |j\rangle$ we obtain a 6×6 matrix which can be diagonalized analytically (see Ref.[1]). In the case of interest to us case we list only the lowest ground–state eigenvalue and corresponding ground states which are

$$\begin{cases} E \equiv \lambda_5 = 2\epsilon + \frac{1}{2}(K+U) + J - \frac{1}{2}D, \\ |\psi_G\rangle = \frac{1}{[D(D-U+K)]^{\frac{1}{2}}}[(4(t+V))\,|4\rangle \\ -(D-U+K)\,|5\rangle], \end{cases} \tag{S2}$$

where parameters are defined in the main text. Their explicit expressions through the matrix elements of the adjustable Slater (atomic) wave functions

$$\begin{cases} \epsilon_a = \beta^2(1+\gamma^2)\epsilon'_a - 2\beta^2\gamma t' \\ t = t_{12} = \beta^2(1+\gamma^2)t' - 2\beta^2\gamma\epsilon'_a, \\ U = \beta^4[(1+\gamma^4)U' + 2\gamma^2 K' - 4\gamma(1+\gamma')V' + 4\gamma^2 J'], \\ K = K_{12} = \beta^4[(1+\gamma^2)K' + 2\gamma^2 U' - 4\gamma(1+\gamma')V' \\ +4\gamma^2 J'], \\ V = V_{12} = \beta^4[\gamma^2)K' + 2\gamma^2 U' - 4\gamma(1+\gamma')V' \\ +(1+\gamma^2)J' \\ J^H \equiv J^H_{12} = J'_{12} = \beta^4[-\gamma(1+\gamma^2)U' \\ -\gamma(1+\gamma^2)K' + (1+6\gamma^2+\gamma^4)V' \\ -2\gamma(1+\gamma^2 J')]. \end{cases} \tag{S3}$$

where β and γ are mixing parameters and ϵ', t', U', K', J', V' are analogous microscopic parameters calculated for the atomic wave functions. They can be expressed explicitly as a

function of interatomic distance R and inverse orbital size α_0 in the Slater form

$$\begin{cases} \epsilon_a' = \alpha_0^2 - 2\alpha_0 - \frac{2}{R} + 2(\alpha_0 + \frac{1}{R})\exp(-2\alpha_0 R), \\ t' = \alpha_0^2 \exp(-\alpha_0 R)[1 + \alpha_0 R + \frac{1}{3}\alpha_0^2 R^2] \\ -4\alpha_0(1 + \alpha_0 R)\exp(-\alpha_0 R), \\ U' = \frac{5}{4}\alpha_0, \\ K' = \frac{2}{R} - \alpha_0 \ \exp(-\alpha_0 R)[\frac{2}{\alpha_0 R} + \frac{3}{2}\alpha_0 R \\ +\frac{1}{3}(\alpha_0 R)^2 + \frac{11}{4}], \\ V' = \alpha_0[\exp(-\alpha_0 R)(2\alpha_0 R + \frac{5}{8\alpha_0 R} + \frac{1}{4}) \\ -\frac{1}{4}(1 + \frac{5}{2\alpha_0 R})\exp(-3\alpha_0 R)], \\ J' = \frac{12}{5R}[S^2 C + S^2 \ \ln(\alpha_0 R) - 2SS' E_i(-2\alpha_0 R) \\ +(S')^2 E_i(-4\alpha_0 R)] \\ +\alpha_0 \ \exp(-2\alpha_0 R)[\frac{5}{4} - \frac{23}{10}\alpha_0 R - \frac{6}{5}\alpha_0^2 R^2 - \frac{2}{15}\alpha_0^3 R^3], \end{cases} \tag{S4}$$

with the Euler constant $C \simeq 0.57722$, and

$$\begin{cases} E_i(x) \equiv -\int_x^\infty \frac{dt}{t} \ \exp(-t), \\ S \equiv \exp(-\alpha R)\left(1 + \alpha R + \frac{1}{3}\alpha^2 R^2\right), \\ S' \equiv \exp(\alpha R)\left(1 - \alpha R + \frac{1}{3}\alpha^2 R^2\right). \end{cases} \tag{S5}$$

In effect, the determination of $|\psi_G\rangle$ requires only readjustment of the Slater-orbital size contained in the expression for Hückel-Wannier orbitals $w_i(\mathbf{r})$, a procedure of which is schematically illustrated by the flowchart in Fig. 4 in the main text.

The microscopic parameters obtained in this way are shown in Figs. S1 and S2. Par-

enthetically, the mixing coefficients β and γ are expressed through the overlap integral $S \equiv \langle \psi_1(\mathbf{r})^\dagger \psi_2(\mathbf{r}) \rangle$

$$\begin{cases} \beta = \frac{1}{\sqrt{2}} \sqrt{\frac{1+\sqrt{1+S^2}}{1-S^2}}, \\ \gamma = \frac{S}{1+\sqrt{1-S^2}}. \end{cases} \tag{S6}$$

The whole procedure is closed once we calculate explicitly the ground-state two-particle

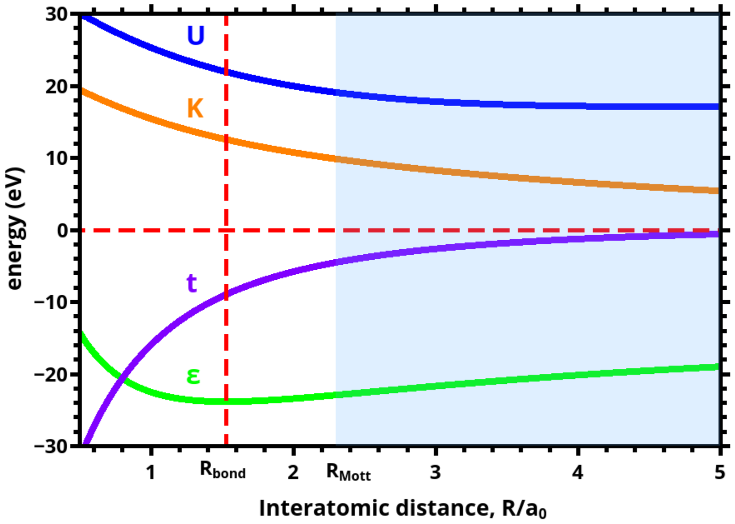

Figure S1: Microscopic parameters indicated versus interatomic distance R. The equilibrium bond length and the onset of *Mottness* (to the right of R_{Mott}) are also marked. The vertical dashed line marks the equilibrium bond length.

wave function according to the general rule

$$\psi_G(\mathbf{r}_1, \mathbf{r}_2) = \frac{1}{2} \langle 0| \{\hat{\psi}_\uparrow(\mathbf{r}_1)\hat{\psi}_\downarrow(\mathbf{r}_2) - \hat{\psi}_\downarrow(\mathbf{r}_1)\hat{\psi}_\uparrow(\mathbf{r}_2)\} |\psi_G\rangle \tag{S7}$$

The final result is the expression (1) in the main text, which contains single-particle molecular wave functions $\{w_i(\mathbf{r})\}_{i=1,2}$. Those wave functions are determined in a separate procedure

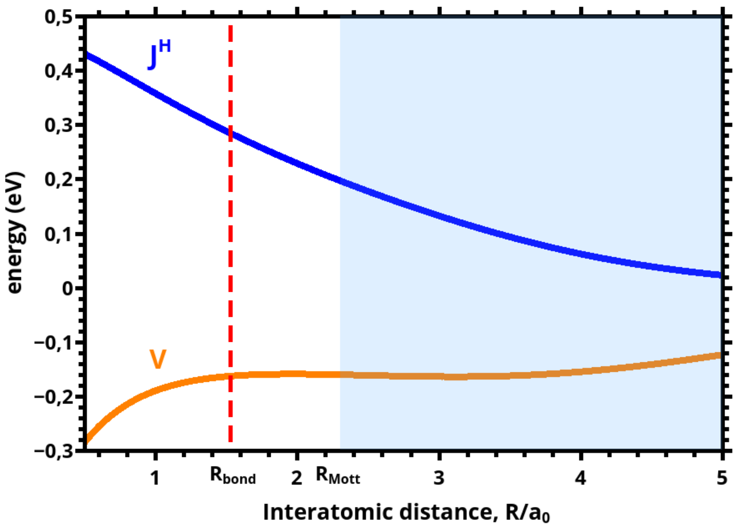

Figure S2: Microscopic parameters, J^H and V versus R (same characteristics as in Fig.(S1)).

described in Sec. Method in main text. The fundamental problem one encounters at the

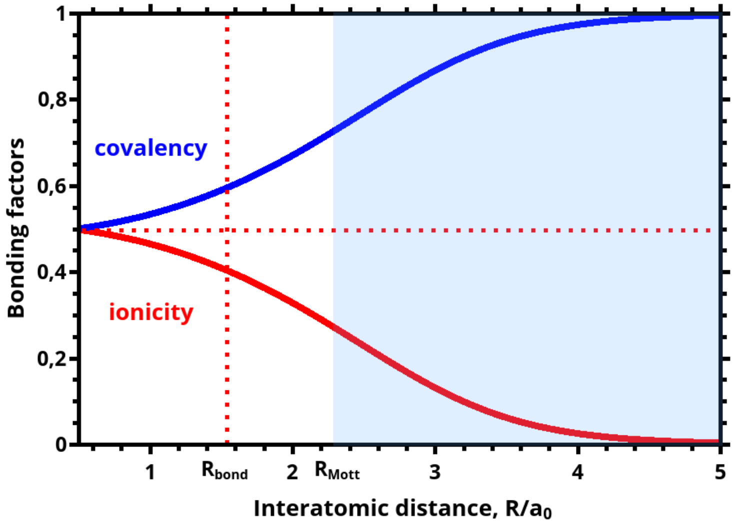

Figure S3: Two-particle covalency and ionicity for H_2 molecule calculated as $|C|^2/(|C|^2 + |I|^2)$ and $|I|^2/(|C|^2 + |I|^2)$, respectively. These results are reinterpreted subsequently and the results, including atomicity, are displayed in Fig. 2 of the main text.

outset is that if one examines the interatomic distance R dependence of the coefficients $|C|^2/(|C|^2 + |I|^2)$ and $|I|^2/(|C|^2 + |I|^2)$; this has been plotted in Fig. S3. Namely, one spots that such naturally defined covalency exhibits a clear unphysical behavior in the limit $R \to \infty$, where the covalency increases, i.e., when the molecular states should reduce to

the parent atomic Slater states. To avoid this basic deficiency the concept of atomicity is introduced, extracted from covalency, and discussed in detail in the main text. Along with the explicit wave function expression (cf. Eqs. (1) and (5)) in main text), one obtains also the corresponding expression for the physical electron densities (cf. Fig 3a-d in the main text). A similar procedure can be applied to more complex types of bonds (e.g., C–C bond, etc.) However, in that situations the explicit expressions replacing those listed in Eqs. (S4) are replaced by the numerical values and the whole analysis presented here, although mathematically feasible, cannot be carried out anymore in analytical terms. We should see further progress along these lines in the near future.

References

(1) Spałek, J.; Podsiadły, R.; Wójcik, W.; Rycerz, A. Optimization of single-particle basis for exactly soluble models of correlated electrons. *Phys. Rev. B* **2000**, *61*, 15676–15687.
