# Supplementary material for: Toward Complementary Characterization of the Chemical Bond
Source: J Phys Chem Lett. 2022 Oct 27;13(44):10261–6. doi: 10.1021/acs.jpclett.2c02544 (PMC9661532; doi:10.1021/acs.jpclett.2c02544)
Supplement: Supplementary file 2 — jz2c02544_si_003.pdf [file jz2c02544_si_003.pdf]

jz-2022-025448.R1

Name: Peer Review Information for "Towards Complementary Characterization of the Chemical Bond"

First Round of Reviewer Comments

Reviewer: 1

Comments to the Author

Hendzel et al. apply both first and second quantization methods to model the two-center two-electron bond of the H_2 molecule. They redefine the covalency and include an atomic contribution, which solves a deficiency in the basic definition. Along with their definitions of ionicity and atomicity factors, this enables a quantitative picture of the chemical bond. These factors agree with Mott and Hubbard criteria, indicating the tendency of localization of the electrons as the interatomic distance increases. This relation is also expressed through definitions of the electron density of the H_2 bond in atomic, ionic and covalent configurations.

This work is a continuation of the authors' previous publication (Maciej Hendzel et al 2022 *J. Phys. B: At. Mol. Opt. Phys.* 55 185101), in which an introduction of the concept of atomicity is given and a physical reinterpretation of atomicity, covalency, and ionicity is provided. In this letter, a further derivation of their previously published method, EDABI, is carried out in order to provide new quantitative definitions of useful properties of the chemical bond of H_2. The good agreement of molecular system properties with Mott-Hubbard features that originate from condensed-matter physics is interesting. Furthermore, figures 1 and 2 are valuable in illustrating the connection between the Mott-Hubbard criteria and the introduced factors. However, the authors do not make any attempt to put this work into context of the state of the art. Only one relevant reference to the history of the chemical bond is included (ref. 12), and not until page 6. Moreover, there is no clear establishment of novelty. The introduction and final summary are vague and lack context. For this reason, I do not recommend this manuscript to be published in *The Journal of Physical Chemistry Letters*.

In addition, the following comments would be useful for the authors to consider:

1. What can you say about the accuracy of the introduced quantities? A comparison with results from another method or experimental data could be useful.

2. There can be issues regarding additivity and/or transferability with wavefunction based methods. It would be useful to mention in what way the introduced quantities can be derived for larger and/or higher bond order systems.

3. The final summary could benefit from examples of applications and/or experimental methods that could take advantage of these results.

4. It would be useful for the expressions for the Mott and Hubbard onset criteria to be more clearly defined.

5. Although no scientific errors were found, I suggest that the language and turns of phrase be reviewed to improve readability.

6. Typo Page 1, Line 28. “the of atomicity”.

7. Typo Page 8, Line 36, Ref. 13. Volume and page numbers are missing.

8. The method section and supporting information correspond to a reiteration of what has already been published. It would be useful to provide more details on what is presented in the main text.

Reviewer: 2

Comments to the Author

This paper proposes a method for the quantification of covalency, iconicity and atomicity of chemical bonds by methods that were initially developed for solids. As shown, in the case of H2, that is the simplest multi-electron molecule,the method yields numerical values for the above characteristics that are consistent with our view and understanding of covalent bonds.

The calculations reported were competently performed and the results are reported in a clear and concise manner.

I recommend publication of this paper in its current form.

Author's Response to Peer Review Comments:

Professor Editor

Senior Editor, The Journal of Physical Chemistry Letters

Dear Professor Editor,

Please find enclosed our revised manuscript (ID: jz-2022-025448) which we would like to resubmit to JPCL. In this version we have revised essentially whole text, with the corrections marked in red in one of the two enclosed coppies of the whole paper.

In particular, we have addressed carefully the question concerning the "novelty and urgency" aspects, which you and Reviewer 1 have raised. Explicitly, we have written a whole Section concerning that aspect in page 2 od the manuscript, as well as have reiterated those in the Recapitulation Section on pp. 6&7. Separately, we also provide a detailed answer of those aspects explicitly in our reply to Reviewer 1. We understand that Reviewer 2 had no requests/queries: we are pleased with his very positive evaluation of our work.

Additionally, regarding points 1-4 of your letter, we have changed our previous version, in agreement with your advise.

Hope, all the corrections are satisfactory.

With kind regards, on behalf of all the authors,

Jozef Spalek

Professor of Physics

Reply to the queries/comments of Reviewer 1

We are pleased with the fair estimate of our work value in the first part of the Review.

What concerns the detailed comments/queries, our reply is as follows. First, in accordance with the Reviewer's suggestion *"to put this work in to context of the state of art"* we have added Sections in pages 1&2, marked in red in the revised version of our manuscript. Furthermore, we have revised essentially the Recapitulation Section on pp. 6&7, also marked in red. We hope that the added text and additional References address properly also the question of *"novelty and urgency"*. In particular, to the best of our knowledge, none of the recent papers quoted has raised the notion of *atomicity*, which is indispensable to recover correctly the atomic limit, where the states are obviously separated. In this manner, one of the fundamental inconsistencies has been removed in theory of the chemical bond. We believe that this factor is universal to correctly define the covalency (see definition of the *true covalency*) *versus* ionicity. We hope that by adding more recent references we have illustrated the fact that the basic questions, concerning the chemical bond nature, is still intensely discussed.

Technical points:

Ad 1. The question of *"accuracy of the introduced quantities"*: Our results are exact within the basis selected in the second quantization. The improvement of the numerical values can come from extension of the single—particle basis when defining the field operators. We have added a paragraph on page 7 (before Section Method) concerning such an extension of our method. We have also added the accuracy of determining the inverse orbital size, cf. right column on page 8. Finally, we have also remarked there on the computational details.

Ad 2. *"Additivity and/or transferability"*: Strictly speaking, we do not understand what the Reviewer means under these terms. However, the two—particle wave function obeys, obviously, the superposition principle, as can be seen from its explicit expression, as it contains superposition of the covalent and ionic parts (see Eqs. (1) and (5)).

As for *"it would be useful to mention in what way the introduced quantities can be derived for larger and/or higher bond order systems"*:

The method can be extended to more complex--bond systems (e.g., C—C bond), when the basis defining the field operators is extended. We should underline, as we believe that the new ingredients introduced here are of sufficient fundamental meaning and therefore, we think that a detailed, more complex numerical analysis, is not necessary here, as it would obscure the principal message of this short paper.

Ad 3. As for *"summary could benefit from examples…":* The results introduce a new universal features of the chemical bond and therefore, should be applicable, in principle, to many systems. We hope, we should be able to see a progress in application of these concepts to real systems in the near future. This is the reason why we think that detailed applications are not needed, or even impossible, to accommodate in such brief Letter.

Ad 4. As for *"… Mott and Hubbard onset criteria to be more clearly defined":* We do not know what the Reviewer means here. They are, in our view, directly defined and explained on pp. 3&4. Furthermore, Figure 1 illustrates directly those criteria. What is really remarkable in our results is that they coincide with the Mott—Hubbard criteria exactly at the point where the *true covalency* and introduced *atomicity* are equal (cf. red point in Fig. 2). This coincidence of criteria provides a direct, if not striking, matching of the condensed—matter—physics and quantum chemistry aspects of the same phenomenon, i.e., the onset of *atomicity* in collective systems.

Ad 5. We have revised the whole text and nitty—gritty things. Hope to the Reader satisfaction.

Ad 6-7. The typos corrected, thanks.

Ad 8. We think that a partial repetition of the Method and Supporting Information Sections was necessary to improve readability/clarity of the concepts presented. However, we have revised both Sections, as marked in red in the present version.

At the end, we would like to thank the Referee for his careful reading of our text.

On behalf of all authors,
Józef Spałek
